# Dr. O’Brien’s Nomination for the Position of Health Officer of the Port of New York

**Published:** 1888-07

**Authors:** 


### *DR. O'BRIEN'S NOMINATION FOR THE POSITION OF HEALTH OFFICER OF THE PORT OF NEW YORK*

During the last session of the late Legislature, the Governor nominated our townsman, Dr. E. C. W. O'Brien, to the position of Health Officer of the port of New York. He was strongly endorsed by the whole medical profession in this city, where he is well known. It was a matter of general congratulation, and his especial fitness for the position was well recognized. The doctor enjoys the reputation of a man of good judgment, and skilled physician. Few doubted that the Senate could reject such a man to fill the position, especially since the term of the present incumbent has expired many years since. But the rumor came to Buffalo that such might be the case. Almost without consultation, a delegation of citizens and physicians was formed to visit Albany and urge the claims of Dr. O'Brien. The delegation went, was given a hearing before a committee, but great was the disappointment of the profession and the public when we learned, for a particular reason, the nomination was rejected. This position has never been given to Buffalo, and on this account, as well as the eminent fitness of the nominee for the position, our disappointment was great. It is to be hoped if the

Senate next year have the opportunity, it will do justice to Dr. O'Brien and this city. The matter, however, was not at all local, as seen by this article from the *New York Medical Journal:*

THE NEW NOMINEE FOR THE HEALTH OFFICERSHIP OF THE PORT.

Dr. Edward C. W. O'Brien, of Buffalo, having been nominated by the Governor for the office of Health Officer of the Port of New York, it is, in our opinion, earnestly to be hoped that the Senate will confirm the nomination. In the matter of the political antagonism that has for so long a time defeated the confirmation of a new officer, this *Journal* has no preferences whatever, as, indeed, it has none in any other affair of pure politics; but it has a decided preference for a suitable incumbent of the important office in question, and such a man it believes Dr. O'Brien to be. The *New York Journal* cites the expressions of opinion by a great number of the medical men of Buffalo, of all shades of political connection and representing both the medical colleges of Buffalo, which we find filling nearly two columns of the Buffalo *Evening News*. All this shows—what we knew well enough before—that Dr. O'Brien is highly esteemed by his fellow-practitioners, those who know him best, and that his services in the great small-pox epidemic in Buffalo, fifteen years ago, when he was the Health Physician of that city, are remembered and appreciated. It is our conviction that there ought to be no delay in his confirmation.

Dr. F. P. Foster, the distinguished editor of the *New York Medical Journal*, has been intimately acquainted with Dr. O'Brien for sixteen years; is, therefore, well informed in regard to Dr. O'Brien's eminent fitness for the responsible office for which Governor Hill nominated him.

